# A novel Killian–Jamieson diverticulectomy using a thyroid gland flap: a case report

**DOI:** 10.1186/s40792-020-01060-z

**Published:** 2020-11-10

**Authors:** Takuya Saito, Tetsuya Ogawa, Shintaro Kurahashi, Hiroki Okamoto, Hirotake Gonda, Tatsuki Matsumura, Takaaki Osawa, Yasuyuki Fukami, Shunichiro Komatsu, Kenitiro Kaneko, Tsuyoshi Sano

**Affiliations:** 1 Department of Gastroenterological Surgery, Aichi Medical University, 1-1 Yazakokarimata, Nagakute, Aichi 480-1195 Japan; 2 Department of Otorhinolaryngology, Aichi Medical University, 1-1 Yazakokarimata, Nagakute, Aichi 480-1195 Japan

**Keywords:** Killian–Jamieson diverticulum, Pharyngoesophageal diverticulum, Thyroid gland flap, Recurrent laryngeal nerve

## Abstract

**Background:**

A Killian–Jamieson diverticulum is a rare pharyngoesophageal diverticulum that is radically treated by diverticulectomy. However, there is no consensus on whether cricopharyngeal myotomy is necessary, and the optimal surgical methods that prevent postoperative complications such as leakage are undetermined.

**Case presentation:**

A 49-year-old man was referred to our hospital with oropharyngeal dysphagia while eating. The patient was preoperatively diagnosed with a Killian–Jamieson diverticulum based on radiographic and clinical findings and underwent a transcervical diverticulectomy. The recurrent laryngeal nerves were preserved using an intraoperative nerve monitoring system, and the diverticulum was identified without difficulty. A partial cricopharyngeal myotomy was performed to expose the base of the diverticulum. The diverticulum was transected transversally using a linear stapler under the guidance of intraoperative upper intestinal endoscopy. A thyroid gland flap supplied by the superior thyroid artery was harvested and placed overlapping the area of the partial cricopharyngeal myotomy. Due to the proximity of the recurrent laryngeal nerve course to the diverticulum stump, the staple line was not buried with sutures. The thyroid gland flap with its rich vascular supply was fixed to completely cover the staple line on the cut surface of the thyroid gland. The postoperative course was uneventful, without vocal cord paralysis. The patient was discharged on postoperative day 8. He developed no clinical signs suggesting leakage, recurrence, or adverse events.

**Conclusion:**

Killian–Jamieson diverticulectomy using a thyroid gland flap and partial cricopharyngeal myotomy is a valid treatment option that may prevent complications and recurrence. Precise evaluation of the diverticulum using an intraoperative nerve monitoring system is crucial for the repair.

## Main text

### Background

Pharyngoesophageal diverticulum is a relatively rare disorder that arises from an anatomically vulnerable area [1]. Three types of pharyngoesophageal diverticula have been identified; Zenker’s diverticulum (ZD) is the most common, followed by Killian–Jamieson diverticulum (KJD) and Laimer’s diverticulum [2]. KJD develops in the anterolateral esophageal wall distal to the cricopharyngeal muscle and presents with specific symptoms, such as dysphagia [3]. Surgery is generally recommended for symptomatic pharyngoesophageal diverticula [4]. Postoperative complications, including esophageal leakage, infection, and stenosis, have been reported for ZD [5]. However, there are no definitive treatment options that effectively prevent postoperative complications. In the field of neck surgery, a thyroid gland flap (TGF) with a sufficient blood supply has been reported to reduce the risk of complications [6]. Surgical diverticulectomy requires an intraoperative neuromonitoring (IONM) system to show the proximity of the recurrent laryngeal nerve (RLN) to the base of the diverticulum. However, there is no consensus regarding whether cricopharyngeal myotomy should be performed in KJ diverticulectomy. We herein report a case in which a KJD was successfully treated with diverticulectomy and partial cricopharyngeal myotomy using a TGF repair without postoperative complications.

### Case presentation

**Fig. 1:**
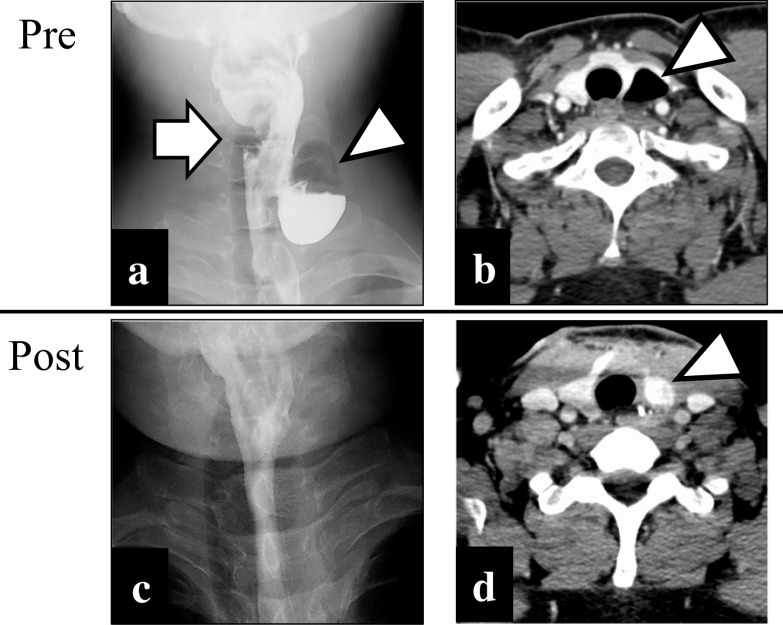
Preoperative and postoperative findings. **a** Preoperative esophagography showing a left-sided outpouching sac just below the cricopharyngeal bar. Arrow: cricopharyngeal bar; arrowhead: diverticulum. **b** Preoperative computed tomography with contrast showing a diverticulum (arrowhead) from the esophagus at the level of the cricothyroid cartilage. **c** Postoperative esophagography reveals no leakage. **d** Postoperative enhanced computed tomography showing that the thyroid gland flap (arrowhead) is accurately placed to cover the defect, and the surgical area is clearly enhanced.

**Fig. 2:**
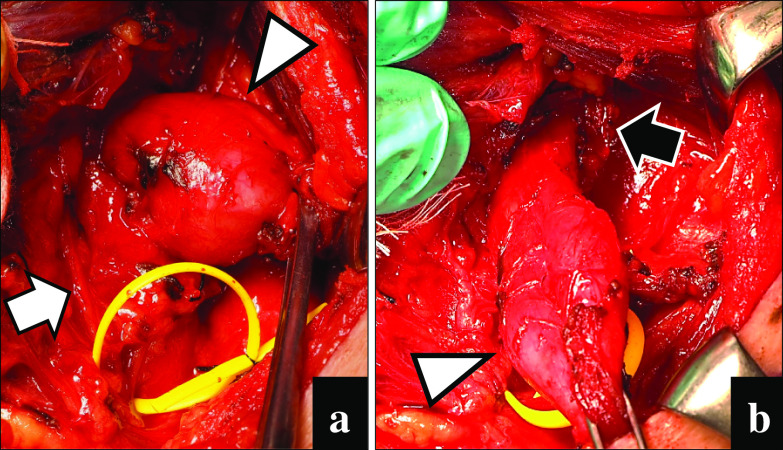
Intraoperative photographs before and after diverticulectomy. **a** A diverticulum (arrowhead) with a defective area on the muscular layer, and the area of the left recurrent laryngeal nerve (white arrow). **b** Intraoperative anatomical relationship between the diverticulum (arrowhead) and cricopharyngeal muscle (black arrow) after partial myotomy

**Fig. 3:**
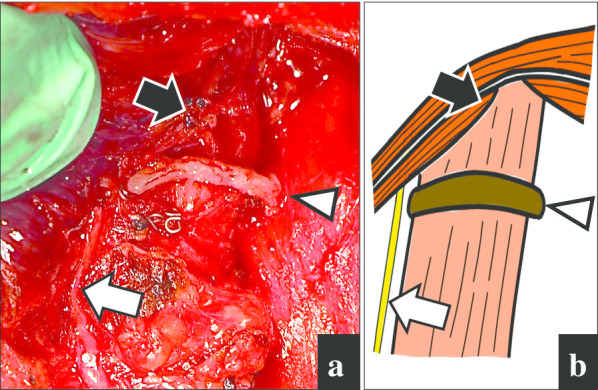
Photograph and illustration showing the positions of the left recurrent laryngeal nerve and transected diverticulum. **a** Area of the left recurrent laryngeal nerve and nearby diverticulum transected transversally after diverticulectomy. White arrow: recurrent laryngeal nerve; arrowhead: staple line; black arrow: cricopharyngeal muscle. **b** Schema of the position between the recurrent laryngeal nerve and transected edge after partial myotomy. White arrow: recurrent laryngeal nerve; arrowhead: staple line; black arrow: cricopharyngeal muscle

**Fig. 4:**
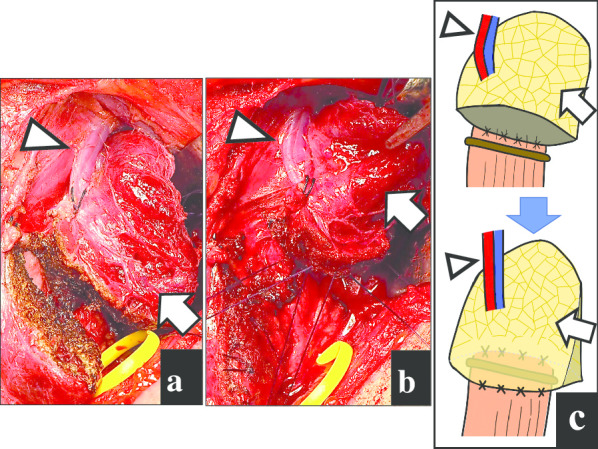
Photographs and illustrations showing the placement of the thyroid gland flap. **a** The thyroid gland flap is cut to fit the area of the diverticulectomy before being placed. **b** The thyroid gland flap is sutured to completely cover the staple line on the cut surface of the thyroid gland. **c** Schema of the staple line covered by the cut surface of the thyroid gland. Arrowhead: superior thyroid artery and vein; arrow: thyroid gland flap

A 49-year-old Japanese man was referred to our hospital with dysphagia for solids and food impaction around the neck for several years. His medical history was otherwise unremarkable, and he had not continuously taken any medications for an extended period. A physical examination revealed no abnormalities, and the results of laboratory tests were within normal ranges. Upper intestinal endoscopy revealed a diverticulum on the left side of the cervical esophagus below the piriform recess (Fig. 1a). There were no mucosal abnormalities or neoplastic lesions in the diverticulum. Barium esophagography showed a left-sided outpouching sac just below the cricopharyngeal bar. This outpouching sac from the cervical esophagus was conspicuous on the front view (Fig. 1b). Contrast-enhanced computed tomography revealed a 35-mm-diameter diverticulum containing gas and food debris behind the left thyroid lobe, connecting to the cervical esophagus at the level of the cricothyroid cartilage (Fig. 1c). Based on these findings, the patient was diagnosed as having a KJD. Transcervical diverticulectomy was performed under general anesthesia. An 8-cm collar skin incision was made two fingers above the sternoclavicular joint with a scalpel. After elevation of the skin flap, the left sternohyoid muscles were preserved. The thyroid gland was exposed, and posterior and lower dissection of the left thyroid lobe was performed. The lower border of the left thyroid lobe was mobilized, and the diverticulum was exposed. The thickened diverticulum was identified without difficulty; however, there was adhesion at the base of the diverticulum. The left RLN was identified and marked for monitoring. The diverticulum was carefully dissected from the surrounding tissue using an IONM system (Nerve Integrity Monitoring Response 3.0 system; Medtronic Japan, Tokyo, Japan) (Fig. 2a). The left RLN is typically located in the tracheoesophageal groove. A handheld probe, which was used to avoid mechanical or thermal injury, revealed that the left RLN was active at the base of the diverticulum. Therefore, it was considered possible to preserve the RLN with a high degree of certainty. The base of the diverticulum was below the cricopharyngeal muscle and lateral to the longitudinal esophageal muscle and was diagnosed as a KJD (Fig. 2b). In definitive diverticulectomy, a partial cricopharyngeal myotomy was performed to expose the cranial side of the base of the diverticulum. The transection line was selected under the guidance of intraoperative upper intestinal endoscopy to ensure both appropriate dissection of the diverticulum and an adequate caliber of the cervical esophageal lumen. The diverticulum was transected transversally using a linear stapler (Fig. 3). Because of the proximity of the RLN course to the diverticulum stump, the staple line was not buried with sutures. To reinforce the staple line, a TGF was prepared. The TGF was directly supplied by the superior thyroid artery and harvested from the superior aspect of the designated thyroid lobe (Fig. 4a). The size of the left thyroid gland was adjusted to fit and then placed on the area of the partial cricopharyngeal myotomy (Fig. 4b). The TGF was fixed so that it completely covered the staple line on the cut surface of the thyroid gland (Fig. 4c). Thus, this weakened area was completely covered with the TGF, which had a rich blood flow from the superior thyroid artery. The total blood loss was 79 mL, and the total surgical time was 153 min. Histopathological examination showed a pseudodiverticulum with a defect of the muscle layer, which was compatible with a KJD. The postoperative course was uneventful, without vocal cord paralysis. Postoperative barium esophagography confirmed that there was no leakage or remnant diverticulum (Fig. 1c). Enhanced computed tomography showed that the TGF was accurately located corresponding to the defect (Fig. 1d). Oral intake was resumed on postoperative day 3, and the patient was discharged on postoperative day 8. He developed no findings suggestive of recurrence or other complications.

## Discussion

The present case highlights two important clinical issues in KJD. One is that diverticulectomy is achievable via a partial cricopharyngeal myotomy, and the other is that a TGF may reduce the risk of postoperative complications.

Suture or staple line leakage following diverticulectomy reportedly occurs in 1.7% to 12.7% of cases [7], and this complication poses a risk of cervical infection and stenosis [5]. Although surgery for KJD is rare, one case report described the occurrence of staple line leakage that was treated by enforced fasting [4]. There is currently no information on the prevention of leakage after diverticulectomy. However, we considered that coating and reinforcing the suture line using a TGF, which is available in the same surgical field, is a minimally invasive and promising method for preventing suture or staple line leakage. Covering the staple line using the sternothyroid muscle is a simple and useful procedure. Swallowing dysfunction can reportedly be caused by defective relaxation of scarred sternohyoid and sternothyroid muscles [8]. Because most chief complaints in patients with KJD are related to swallowing dysfunction, a TGF is an option for this procedure. The use of a TGF for glottic reconstruction after vertical laryngectomy was initially reported in 1990 by Kojima et al. [9]. Since then, the effectiveness of a TGF has been demonstrated in larynx-preserving pharyngectomy and supracricoid partial laryngectomy after radiochemotherapy [6, 10]. Furthermore, Ogawa et al. [6] showed that a TGF with a sufficient blood supply prevents cervical infection and dehiscence. We harvested a TGF from the superior aspect of the designated thyroid lobe. The TGF had an adequate blood supply via the superior thyroid artery, which is the first branch of the external carotid artery, and was smoothly rotated and moved to the staple line area. The staple line was not buried because of the proximity of the RLN course to the diverticulum stump. Therefore, we reinforced the staple line by suturing to completely cover it on the cut surface of the TGF. The TGF is also less likely to shrink than other flaps because of its abundant blood supply [9]. In the event of cervical infection, the area of the diverticulectomy is adequately filled, and this blood supply ensures high local drug concentrations, thereby preventing further complications. However, because a previous study showed the potential for postoperative thyroid enlargement, respiratory disturbances need to be considered [11]. No complications were observed in the present case, and respiratory disturbances were unremarkable. Furthermore, there was no thyroid swelling caused by congestion because the superior thyroid vein is located lateral to the artery. If the parathyroid glands are removed at the same time as formation of the TGF, the diverticulum resection area is filled with the TGF containing the parathyroid glands. For the safety of the patient, the serum calcium level should be measured several times postoperatively to confirm that there are no abnormalities.

Whether partial cricopharyngeal myotomy is adequate for KJ diverticulectomy remains controversial. We consider that myotomy provides a sufficient surgical field in which it is safe to perform diverticulectomy after identification of the RLN. Appropriate diverticulectomy at the base may prevent recurrence of the diverticulum. The gold standard for the surgical treatment of a ZD is complete cricopharyngeal myotomy using an external approach. However, because of the risk of postoperative cricopharyngeal muscle dysfunction, we consider it preferable to perform an incomplete (approximately half) cricopharyngeal myotomy that exposes the base of the diverticulum. Transcervical diverticulectomy should be performed using an IONM system because of the high risk of injury to the RLN, which enters the neck at the base of the diverticulum [12]. Because the RLN is located behind the cricopharyngeal muscle, IONM systems are also useful in cricopharyngeal myotomy at the cranial side of the diverticulum. Early localization and identification of the RLN was possible in the present case, and the diverticulum was easily dissected from the surrounding tissue in a short period. It may be difficult to define the RLN when the KJD is associated with chronic inflammatory changes [13]. The diverticulum is often resected at its base using a linear stapler. Although a linear stapler is generally used longitudinally, we transected the diverticulum transversally under the guidance of upper intestinal endoscopy [12]. A sufficient surgical field after myotomy facilitates reliable transverse dissection to the cranial side of the diverticulum.

Our method raises concerns about thyroid function and disease, such as chronic thyroiditis and Graves’ disease; therefore, preoperative morphological and functional assessments of the thyroid gland are mandatory. Even in euthyroid patients, preoperative cervical ultrasonography should be performed to confirm the absence of thyroid gland abnormalities. Testing of perioperative thyroid function is also important. Anatomical variations in the location of the RLN have been reported for different diverticula [12, 14, 15]. The anatomical position of the diverticulum may affect the methods required to diagnose the cause of oropharyngeal dysphagia [14]. Barium esophagography often establishes the diagnosis of a pharyngoesophageal diverticulum. A KJD is detected on the lateral wall of the pharyngoesophageal junction and below the cricopharyngeal muscle (cricopharyngeal bar) on the anteroposterior view [16]. Because of its proximity to the thyroid gland, a KJD may be misdiagnosed as a thyroid lesion or nodule. On computed tomography, it is important to distinguish between a cystic lesion and an air-containing cystic lesion originating from the esophagus anterolaterally [17, 18].

## Conclusion

The performance of a partial cricopharyngeal myotomy for adequate KJ diverticulectomy and subsequent TGF repair is a promising method that may reduce the risks of postoperative complications and disease recurrence. The preoperative investigation of thyroid function and disease as well as the use of an IONM system are critical for this surgical option.

## Data Availability

Data sharing is not applicable to this article as no datasets were generated or analyzed during the current study.

## Abbreviations

ZD: Zenker’s diverticulum
KJD: Killian–Jamieson diverticulum
KJ: Killian–Jamieson
RLN: Recurrent laryngeal nerve
TGF: Thyroid gland flap
IONM: Intraoperative nerve monitoring
